# Low Sulfur Amino Acid, High Polyunsaturated Fatty Acid Diet Inhibits Breast Cancer Growth

**DOI:** 10.3390/ijms24010249

**Published:** 2022-12-23

**Authors:** Riccardo Turchi, Flavia Tortolici, Monica Benvenuto, Carolina Punziano, Anastasia De Luca, Stefano Rufini, Raffaella Faraonio, Roberto Bei, Daniele Lettieri-Barbato, Katia Aquilano

**Affiliations:** 1 Department of Biology, University of Rome Tor Vergata, 00133 Rome, Italy; 2 Departmental Faculty of Medicine, Saint Camillus International University of Health and Medical Sciences, 00131 Rome, Italy; 3 Department of Clinical Sciences and Translational Medicine, University of Rome Tor Vergata, 00133 Rome, Italy; 4 Department of Molecular Medicine and Medical Biotechnologies, University of Naples Federico II, 80131 Naples, Italy; 5 IRCCS Santa Lucia, 00179 Rome, Italy

**Keywords:** NRF2, p53, lipid peroxidation, mitochondria, ferrostatin-1, ferroptosis

## Abstract

Cancer cells may acquire resistance to stress signals and reprogram metabolism to meet the energetic demands to support their high proliferation rate and avoid death. Hence, targeting nutrient dependencies of cancer cells has been suggested as a promising anti-cancer strategy. We explored the possibility of killing breast cancer (BC) cells by modifying nutrient availability. We used in vitro models of BC (MCF7 and MDA-MB-231) that were maintained with a low amount of sulfur amino acids (SAAs) and a high amount of oxidizable polyunsatured fatty acids (PUFAs). Treatment with anti-apoptotic, anti-ferroptotic and antioxidant drugs were used to determine the modality of cell death. We reproduced these conditions in vivo by feeding BC-bearing mice with a diet poor in proteins and SAAs and rich in PUFAs (LSAA/HPUFA). Western blot analysis, qPCR and histological analyses were used to assess the anti-cancer effects and the molecular pathways involved. We found that BC cells underwent oxidative damage to DNA and proteins and both apoptosis and ferroptosis were induced. Along with caspases-mediated PARP1 cleavage, we found a lowering of the GSH-GPX4 system and an increase of lipid peroxides. A LSAA/HPUFA diet reduced tumor mass and its vascularization and immune cell infiltration, and induced apoptosis and ferroptotic hallmarks. Furthermore, mitochondrial mass was found to be increased, and the buffering of mitochondrial reactive oxygen species limited GPX4 reduction and DNA damage. Our results suggest that administration of custom diets, targeting the dependency of cancer cells on certain nutrients, can represent a promising complementary option for anti-cancer therapy.

## 1. Introduction

Cancer cells have the ability to evade regulated forms of cell death such as apoptosis, necroptosis or autophagy [1]. Ferroptosis is the most recently discovered type of regulated cell death characterized by the iron-dependent accumulation of lipid peroxidation products that is antagonized by several antioxidant defense systems including the glutathione peroxidase 4 (GPX4) [2]. Importantly, many cancer cells, such as breast cancer (BC) cells, which escape other means of killing or develop chemoresistance, may be selectively sensitized to ferroptosis [3,4,5]. Therefore, in recent years, therapeutic applications aimed at triggering ferroptosis in cancer treatment has received increasing attention [6,7]. 

Metabolic reprogramming is one of the first distinguishing features of cancer cells. Alteration of central carbon metabolism (glycolysis, tricarboxylic acid cycle and pentose phosphate pathway) has been extensively studied. However, recently, non-carbon metabolism has received increasing attention. Amino acids play important roles in cancer metabolism and experience reprogrammed metabolism in cancer cells as they serve in several cancer cell activities [8]. Actually, amino acids can be used as alternative fuel sources (e.g., glutamine) or as substrates for biosynthetic pathways such as lipogenesis (e.g., branched chain amino acids) and nucleotide synthesis (e.g., glycine, serine) [8]. 

Cancer cell metabolism relies highly on the sulfur amino acids (SAAs) methionine and cysteine, as they participate in several redox reactions that yield the energy and biomass required for tumor growth. SAAs also facilitate the epigenetic regulation of gene expression and provide intermediate metabolites, constituting the cellular antioxidant system, all of which are required for tumor progression [9]. Importantly, SAA restriction in cancer may impair the glutathione (GSH)/GPX4 system, thus increasing the susceptibility to iron-mediated lipid peroxidation and in turn to ferroptosis [10]. As GSH is an important co-factor of GPX4 activity, GSH depleting drugs, such as those that reduce cysteine availability or GSH synthesis (e.g., erastin, L-buthionine sulfoximine), efficiently promote ferroptosis and even enhance the effectiveness of standard anti-cancer drugs [7,11]. In line with this evidence, SAA restriction is effective in repressing cancer growth and improving standard cancer therapies [12,13,14].

Dietary polyunsaturated fatty acids (PUFAs) have been shown to have preventive or even anti-cancer effects and their supplementation is considered a valid pro-ferroptotic and anti-cancer approach, thanks to their capacity to propagate lipid peroxidation cascade [15,16,17]. Based on the effectiveness of either SAA restriction or PUFA supplementation in inducing ferroptosis, in this work, we explored the option to use a dietary approach consisting of combining SAA depletion with PUFA overload to inhibit tumor progression in a mouse model of BC. We found that this nutritional strategy significantly restrains tumor mass expansion by limiting angiogenesis, epithelial to mesenchymal transition, immune cell infiltration and by promoting damage to DNA as well as lipid peroxidation.

## 2. Results

### 2.1. Human Breast Cancer Cells Are Susceptible to Apoptosis and Ferroptosis upon Nutrient Changes In Vitro

**Figure 1:**
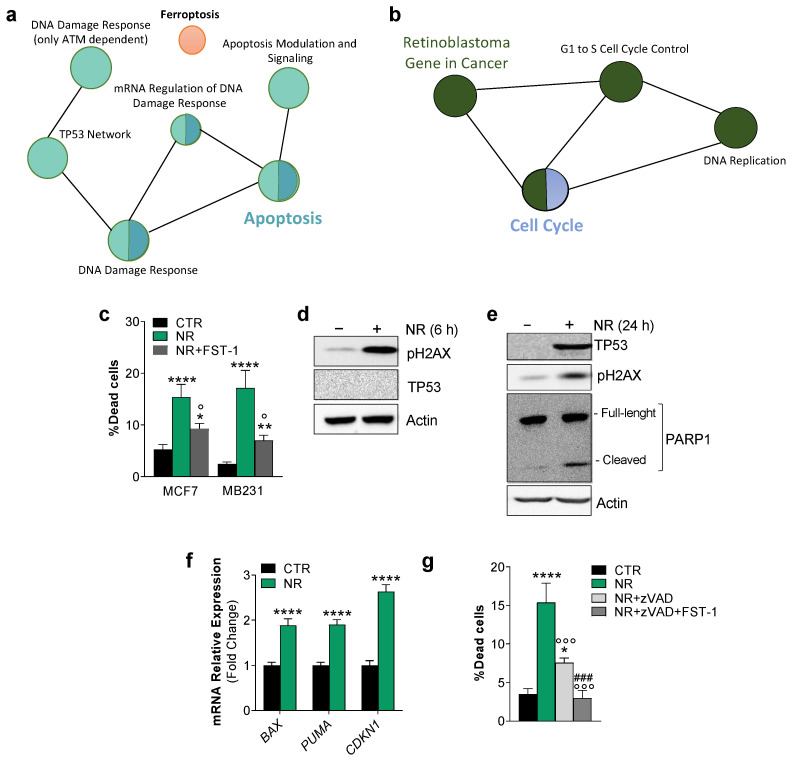
Breast cancer cells undergo both apoptosis and ferroptosis upon nutrient restriction. (a,b) Functional enrichment analysis of microarray data (GSE121378) using Cytoscape software. Wikipathways of the top 200 up-regulated (**a**) and down-regulated (**b**) genes (FC > 1.5, FC < 0.5; *p* < 0.05) are reported. (**c**) Percentage of dead breast cancer cells after 24 h nutrient restriction (NR: HBSS, 1 g/L glucose, 1% FBS) with or without 1 h pre-treatment with 5 μM ferrostatin-1 (FST-1). (**d**,**e**) Western blot analysis of pH2AX, p53, and PARP-1 on MCF7 cells treated with NR. Actin was used as loading control. Immunoblots reported are representative of three independent experiments yielding similar results. (**f**) RT-qPCR analysis of the mRNA expression of p53 downstream genes BAX, PUMA, CDKN1. (**g**) Percentage of dead breast cancer cells after 24 h NR with or without 1 h pre-treatment with 10 μM z-VAD-fmk (zVAD) and 5 μM FST-1. Data are expressed as mean ± S.D. (*n* = 3; **** *p* < 0.0001, ** *p* < 0.01, * *p* < 0.05, vs. CTR; ° *p* < 0.05, °°° *p* < 0.001 vs. NR; ^###^ *p* < 0.001 vs. NR and NR + zVAD).

We previously demonstrated that nutrient restriction (NR), or fasting, modulates ferroptosis hallmarks in muscle cells [18]. Modulating nutrient availability is a promising approach to selectively killing cancer cells, to prevent cancerogenesis and augment the efficacy of anti-cancer therapies [19]. However, the pathways involved in such beneficial effects are still far from being fully elucidated. To have a more comprehensive picture of the BC cell responses to NR, we firstly analyzed a free available microarray dataset (GSE121378) obtained from MCF7 cells grown upon NR conditions. Enrichment analysis of differentially expressed genes performed against Wikipathways showed an up-regulation (FC > 1.5, *p* < 0.05) of 7 genes pertaining to the TP53 (p53) network, 14 genes to DNA damage response, 17 genes to Apoptosis and 8 genes to Ferroptosis (Figure 1a). Wikipathways enrichment results also revealed the downregulation (FC < 0.5, *p* < 0.05) of 19 genes annotated in Cell Cycle and 15 genes in G1 to S cell cycle control pathways (Figure 1b). We hence cultured MCF7 and MDA-MB-231 (MB231) human BC cell lines under similar NR conditions (HBSS, 1% FBS) to investigate the anti-proliferative effects of NR more deeply. Cell viability was analyzed after 24 h and a significant increase of dead cells was observed in both cell lines (Figure 1c). By pre-treating MCF7 and MB231 cells with the ferroptosis inhibitor ferrostatin-1 (FST-1), we found that NR-mediated cell death was significantly decreased (Figure 1d), demonstrating the induction of ferroptosis. However, FST-1 did not completely abrogate cell death, arguing that another type of cell death, likely apoptosis, could occur concomitantly.

DNA damage and cell cycle G1 arrest result in commitment to death by means of apoptosis and/or ferroptosis [20]. As p53 is an important hub involved in cell cycle arrest and repair of DNA double-stranded breaks as well as in ferroptosis induction [21], we firstly evaluated p53 along with DNA damage and apoptosis marks in NR-treated MCF7 cells. NR induced the maximum level of pH2AX accumulation as early as 6 h (Figure 1d). H2AX exerts a key role in promoting p53/p21-mediated cell cycle arrest after DNA replication stalling [22]. p53 was undetectable at 6 h from NR administration, whereas it was found increased at 24 h (Figure 1e). Accordingly, at 24 h of NR treatment in MCF7 cells, we found increased mRNA levels of its target genes such as Puma, Bax and Cdkn1 (alias p21) (Figure 1f). We also found a decrease in the full-length PARP1 protein, an enzyme that is involved in DNA repair, and an increase in its inactive cleaved form (Figure 1e) derived from caspase-3,-7 activity during the execution phase of apoptosis [23]. Pre-treatment with the caspase inhibitor zVAD significantly but not completely abrogated cell death (Figure 1g), confirming the concomitant induction of apoptosis and ferroptosis upon NR.

**Figure 2:**
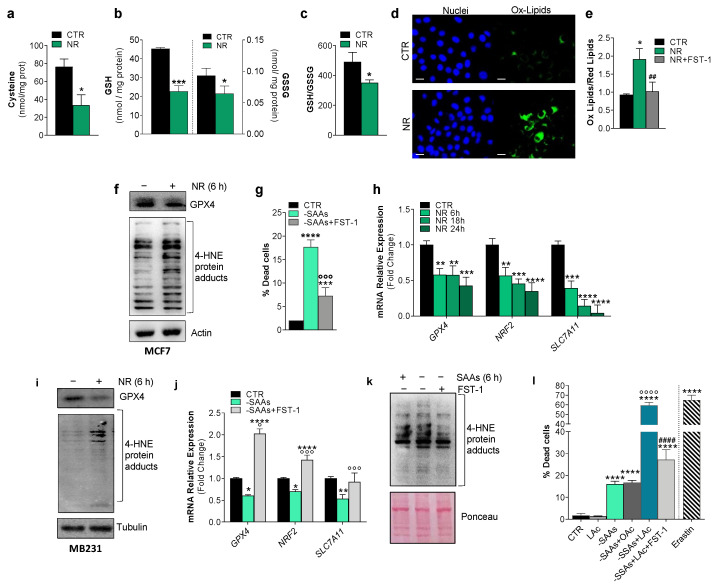
Nutrient restriction modulates typical ferroptotic hallmarks in breast cancer cells. (**a**–**c**) MCF7 cells were subject to nutrient restriction (NR: HBSS, 1 g/L glucose, 1% FBS) for 6 h. Intracellular cysteine (**a**), reduced (GSH) and oxidized (GSSG) glutathione (**b**) were measured by HPLC analysis and redox state was evaluated by calculating GSH/GSSG ration (**c**). (**d**,**e**) Lipid peroxides were determined by means of fluorescent microscopy (**d**) or cytofluorimetric (**e**) analysis after staining with the lipid oxidation sensitive probe C11-BODIPY 581/591. Scale bar: 20 μm. (**f**) Western blot analysis of GPX4 and 4-HNE-protein adducts in MCF7 cells cultured under NR conditions for 6 h. Actin was used as a loading control. (**g**) Percentage of dead MCF7 cells after 24 h sulfur amino acid depletion (-SAAs) with or without 1 h pre-treatment with 5 μM ferrostatin-1 (FST-1). (**h**) RT-qPCR analysis of the mRNA expression of the ferroptosis-related genes GPX4, NRF2 and SLC7A11 in MCF7 cells upon NR. (**i**) Western blot analysis of GPX4 and 4-HNE-protein adducts in MB231 cells cultured under NR conditions for 6 h. Tubulin was used as loading control. (**j**) RT-qPCR analysis of the mRNA expression of the ferroptosis-related genes GPX4, NRF2 and SLC7A11 in MB231 cells after 6 h SAAs depletion with or without 1 pre-treatment with 5 μM FST-1. (**k**) Western blot analysis of 4-HNE-protein adducts in MB231 cells after 6 h SAAs depletion with or without 1 pre-treatment with 5 μM FST-1. Ponceau red was used as loading control. Immunoblots reported are representative of three independent experiments yielding similar results. (**l**) Percentage of dead MCF7 cells after 24 h SAAs depletion with or without 400 μM linoleic acid (LAc) or with or without 1 pre-treatment with 5 μM FST-1. Erastin (10 μM) treatment was used as positive control of ferroptosis. Data are expressed as mean ± S.D. (*n* = 4; **** *p* < 0.0001, *** *p* < 0.001, ** *p* < 0.01, * *p* < 0.05 vs. CTR; °°°° *p* < 0.0001, °°° *p* < 0.001, ° *p* < 0.05 vs. -SAAs; ^####^ *p* < 0.0001 vs. -SAA + LAc; *^##^ p* < 0.01 vs. NR).

Cysteine depletion leads to deficit in the synthesis of glutathione, the main non-enzymatic thiol antioxidant and anti-ferroptotic agent assisting the removal of lipid peroxides by GPX4 [10]. Given the key role of cysteine in modulating ferroptosis, the obtained results prompted us to evaluate whether intracellular cysteine lowering could be the primary mediator of ferroptosis induction upon NR. We firstly evaluated the intracellular content of cysteine and the cysteine containing tripeptide glutathione in MCF7 cells upon NR. Intracellular cysteine (Figure 2a) as well as reduced (GSH) and oxidized (GSSG) glutathione levels were decreased (Figure 2b) with the GSH/GSSG ratio shifting towards pro-oxidant conditions (Figure 2b). By monitoring the amount of lipid peroxides by fluorescent microscopy and cytofluorimetric analysis, using a specific fluorescent probe, as soon as 6 h after NR treatment, we observed a significant increase of such species in MCF7 cells (Figure 2d,e), indicative of the occurrence of iron-mediated oxidative damage to lipids. In line with this assumption, we found that FST-1 treatment was able to completely prevent lipid oxidation (Figure 2e). We also detected an increase of 4-HNE-protein adducts (Figure 2f), which represent irreversible protein oxidation products that are generated when the rate of lipid peroxides overcomes the GSH/GPX4 antioxidant defense. These data prompted us to evaluate whether GPX4 could be affected by NR and we found that its protein content was downregulated (Figure 2f).

To more convincingly demonstrate the specific sensitivity of BC cells to cysteine lowering upon NR, we evaluated cell death in MCF7 cells incubated in a culture medium lacking sulfur amino acid (-SAAs), i.e., cysteine and its precursor methionine. Expectedly, the absence of SAAs induced cell death by means of ferroptosis as demonstrated by the ability of its inhibitor FST-1 to decrease the percentage of dead cells (Figure 2g). Similar data were obtained in MB231 cells. 

Beyond its role in promoting apoptosis, p53 can induce ferroptosis by repressing the transcription of SLC7A11 [24], thus creating a favorable oxidized environment for ferroptosis execution. p53 and Nuclear Factor Erythroid 2-Related Factor 2 (NRF2) are hub genes in the biology of ferroptosis and at the same time govern the adaptive response to environmental stressors including nutrient changes [25,26]. Interestingly, in line with the observed decreased cysteine and glutathione levels upon NR, along with the expected decrease of GPX4 mRNA, we found a significant and progressive downregulation of the cystine/glutamate antiporter xCT (SLC7A11) mRNA (Figure 2h). SLC7A11 is a gene target of both p53 and NRF2 with p53 repressing and NRF2 inducing its mRNA expression [24]. NRF2 also induces the expression of genes pertaining to glutathione metabolism [18]. Expectedly, we observed a decrease of NRF2 mRNA in NR-treated cells (Figure 2h). The modulation of ferroptosis markers, such as reduction of GPX4 protein and accumulation of 4-HNE-protein adducts by NR was also confirmed in MB231 cells (Figure 2i). As for NR, in MB231 cells upon SAA deprivation, we found a decrease of the mRNA levels of SLC7A11, NRF2 and GPX4 genes that was recovered by FST-1 pre-treatment (Figure 2j). In line with these results, the levels of 4-HNE protein adducts increased upon SAA depletion, and FST-1 treatment prevented such an event in MB231 cells (Figure 2k). Similar results were obtained in MCF7 cells.

Finally, to further enhance ferroptosis, concomitant to SAA deprivation, we treated MCF7 cells with n-6 linoleic acid (LAc), a high oxidizable PUFA that is able to propagate a lipid peroxidation cascade, characterizing ferroptosis. As shown in Figure 2l, such a combined approach gave rise to a significant increase of cell death with respect to SAA depletion alone and to levels comparable to those obtained by treating MCF7 cells with the well-known ferroptosis inducer erastin. Moreover, as expected, co-treatment with the saturated fatty acid oleic acid did not give rise to enhancement of cell death, and FST-1 significantly counteracted cell death induced by SAA deprivation combined with LAc treatment (Figure 2l). The same results were obtained in MB231 cells.

### 2.2. A Low SAA-High PUFA Diet Modulates Ferroptosis Marks and Restrains Breast Cancer Growth in Mice

**Figure 3:**
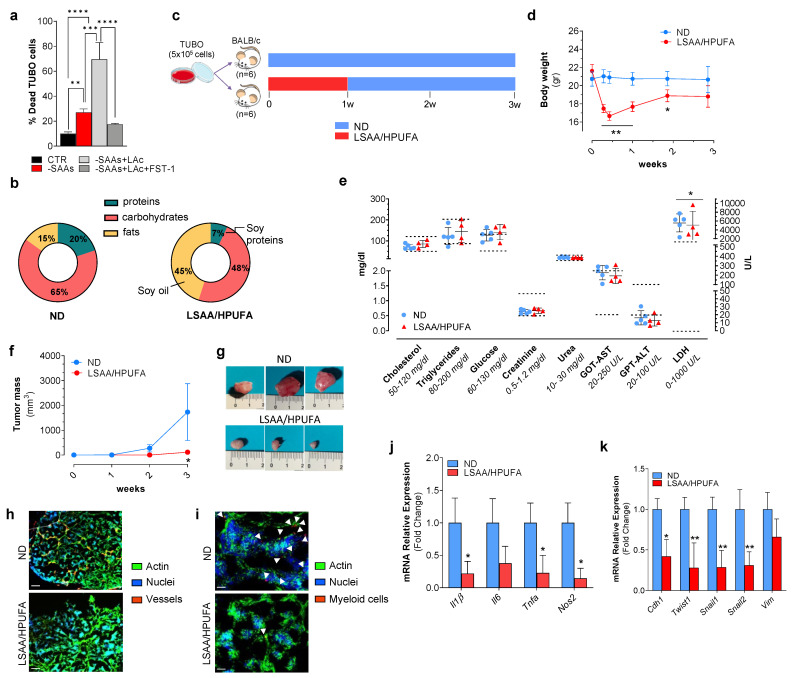
Reducing sulfur amino acids (SAAs) and increasing peroxidizable polyunsaturated fatty acids (PUFAs) through diet counteracts breast cancer development in mice. (**a**) Percentage of dead TUBO cells after 24 h SAAs depletion with or without 400 μM linoleic acid (LAc). Ferrostatin-1 (FST-1) was used at 5 μM concentration and added 1 h prior treatment. Data are expressed as mean ± S.D. (*n* = 4, ** *p* < 0.01, *** *p* < 0.001, **** *p* < 0.0001). (**b**,**c**) Schematic representation of normal (ND) and low SAA-high PUFA (LSAA/HPUFA) diet composition (**b**) and experimental design of the in vivo study (**c**). (**d**) Body weight was evaluated weekly until to sacrifice (** *p* < 0.01, * *p* < 0.05). (**e**) Analysis of bio-clinical parameters after 3 weeks from TUBO cell injection in mice. Dashed lines indicate the range of normal values. Data are expressed as mean ± S.D. (*n* = 5 mice ND group; *n* = 4 mice LSAA/HPUFA group; * *p* < 0.05 vs. ND). (**f**) Tumor masses were evaluated weekly until to sacrifice. (**g**) Representative photographs of explanted breast cancer masses. (**h**,**i**) Representative immunofluorescence images of intratumor vessels (**h**) and immune cell infiltrates (**i**) detected by using Von Willebrand and S100A9 antibody, respectively. Incubation with AlexaFluor488-conjugated phalloidin and Hoechst 33342 were used to visualize actin cytoskeleton and nuclei, respectively. White arrows in (**i**) indicate the presence of myeloid cells within the tumor mass. Scale bar: 100 μm. (**j**,**k**) RT-qPCR analysis of the mRNA expression of the inflammation-related genes Il1b, Il6, Tnfa and Nos2 (**j**) and epithelial to mesenchymal transition genes Cdh1, Twist1, Snail1, Snail2 and Vim (**k**). All the images reported are representative of *n* = 6 mice/group. Data are expressed as mean ± S.D. (*n* = 9 mice/group; ** *p* < 0.01, * *p* < 0.05 vs. ND).

Overall, the in vitro data highly supported the idea that nutrient modulation could efficiently promote cell death in BC cells. Hence, we moved on recapitulating in vitro experiments in a murine model of BC consisting in Balb/c mice subcutaneously inoculated with a Balb/c-derived BC cell line (i.e., TUBO cells). Before starting the in vivo experiment, we verified whether SAA deprivation was able to induce ferroptosis in these cells and LAc to exacerbate this event. As shown in Figure 3a, cell death was increased in TUBO cells, and FST-1 treatment was able to reduce the number of dead cells. To reproduce ferroptosis in vivo, immediately after inoculation, mice were subject to a feeding regimen with a diet containing lower amounts of proteins (poor in SAAs) and carbohydrates, and higher content of PUFA (LSAA/HPUFA) with respect to normal diet (ND) (Figure 3b). Importantly, this diet was a LAc-enriched diet as fats were from soy oil (as reported in CHEBI:166975—soybean oil). After 1 week, the LSAA/HPUFA diet was replaced with ND for 2 weeks (Figure 3c). Body weight was monitored throughout the duration of the experiment (3 weeks). Body weight underwent a reduction in mice fed with LSAA/HPUFA and was recovered later without any effects on bio-clinical parameters with respect to mice under ND (Figure 3d,e), indicating the absence of toxic effects of LSAA/HPUFA. Tumor masses were not palpable after 1 week; at week 2, they tended to be smaller in the LSAA/HPUFA with respect to the ND group and became significantly reduced at 3 weeks (Figure 3f). 

Based on these data, we sacrificed mice after 3 weeks and tumor masses were explanted. Phenotypically, tumor masses explanted from the LSAA/HPUFA group were smaller and apparently less vascularized than those from the ND mouse group (Figure 3g). An immunofluorescence analysis on tumor tissue sections using the Von Willebrand Factor evidenced the complete absence of this endothelial marker in LSAA/HPUFA-fed mice (Figure 3h), indicating the inhibited angiogenesis. Local inflammation due to infiltration of immune cells in tumor masses supports cancer malignancy [27]. Interestingly, myeloid cells infiltrated within the BC mass were markedly reduced in LSAA/HPUFA-fed mice, as assessed by staining with a S100A9 antibody (Figure 3i), and accordingly pro-inflammatory Nos2 and cytokine mRNAs (i.e., Tnfa, Il1b) were significantly up-regulated (Figure 3j). The epithelial–mesenchymal transition (EMT), first described in embryonic development, is one of the main mechanisms involved in BC progression and metastasis [28,29,30]. The analysis of the mRNA expression of some well-known EMT markers evidenced their down-regulation in BC masses explanted from mice fed with the LSAA/HPUFA diet (Figure 3k).

**Figure 4:**
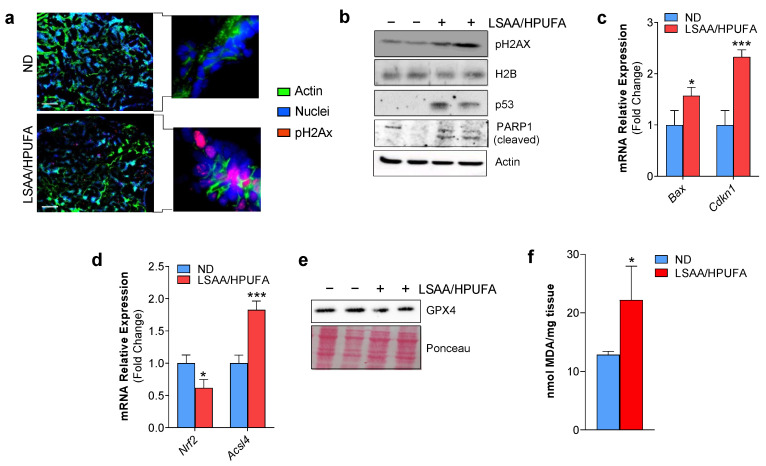
Reducing sulfur amino acids (SAAs) and increasing peroxidizable polyunsaturated fatty acids (PUFAs) through diet induces apoptosis and ferroptosis in breast cancer. (**a**) Representative immunofluorescence images of nuclear DNA damage detected by using pH2AX antibody in explanted breast cancer masses from low SAA–high PUFA (LSAA/HPUFA) or normal diet (ND) group. Incubation with AlexaFluor488-conjugated phalloidin and Hoechst 33342 were used to visualize actin cytoskeleton and nuclei, respectively. Scale bar: 100 μm. (**b**) Western blot analysis of pH2AX, p53 and PARP1 in explanted tumor masses. H2B and Actin were used as loading controls. (**c**,**d**) RT-qPCR analysis of the mRNA expression of the p53 downstream apoptosis-related genes Bax and Cdkn1 (**c**) and ferroptosis-related genes Nrf2 and Acsl4 (**d**). (**e**) Western blot analysis of GPX4 in explanted tumor masses. Ponceau red staining was used as loading control. (**f**) Measurement of malondialdehyde (MDA) levels in explanted tumor masses through a colorimetric assay. All the immunoblots reported are from *n* = 2 mice out of *n* = 6 mice/group yielding similar results. Data are expressed as mean ± S.D. (*n* = 9 mice/group; *** *p* < 0.001, * *p* < 0.05 vs. ND).

We then decided to evaluate whether the LSAA/HPUFA diet, designed to modulate ferroptotic markers in BC, was also able to cause DNA damage and apoptosis in a way similar to in vitro conditions. In sharp contrast with the ND fed group, through pH2AX immunostaining, we found many pH2AX-positive nuclear foci interspersed in the tumor mass explanted from LSAA/HPUFA-fed mice (Figure 4a), indicative of the capacity of the LSAA/HPUFA diet to provoke a persistent DNA damage to BC cells in vivo as well. To validate this result, we performed Western blot analyses on tumor mass homogenates and, in line with the in vitro data, we observed the increase of pH2AX (Figure 4b). This was accompanied by the up-regulation of the p53 protein and its downstream apoptosis-related genes (Figure 4c) as well as by the decrease of full-length PARP1 and appearance of its cleaved products (Figure 4b), overall demonstrating the occurrence of DNA damage-mediated cell growth arrest and apoptosis. Concerning the ferroptosis hallmarks, the mRNA of the anti-ferroptosis transcription factor NRF2 and the protein level of GPX4 were downregulated, and the pro-ferroptotic acyl-CoA synthetase long-chain family member 4 (Acsl4) was up-regulated by the LSAA/HPUFA diet in tumor masses (Figure 4d,e), indicating an impaired antioxidant response and increased incorporation of peroxidizable lipids in cell membranes. Moreover, the occurrence of ferroptosis was finally confirmed by assaying the level of malondialdehyde (MDA), a peroxidation byproduct of cell membrane lipids, that resulted in significantly higher BC masses explanted from LSAA/HPUFA with respect to the ND mouse group (Figure 4f).

### 2.3. Ferroptosis in Breast Cancer Cells Is Mitochondria-Dependent

**Figure 5:**
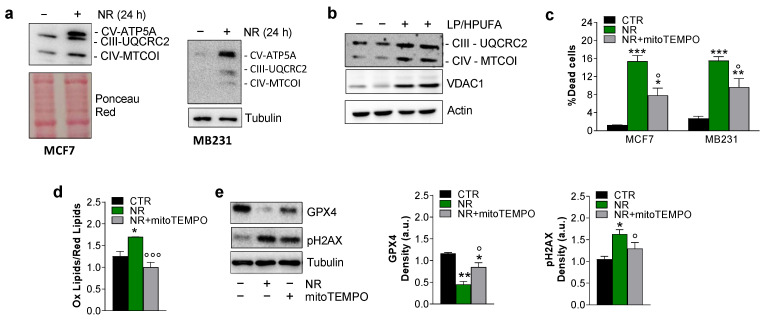
Ferroptosis is induced in a mitochondria-dependent manner in cancer cells. (**a**,**b**) Mitochondrial mass was evaluated by Western blot analysis of electron transport chain subunits and/or VDAC1 in breast cancer cell lines cultured under 24 h nutrient restriction (NR) (**a**) or breast cancer masses explanted from mice fed with LSAA/HPUFA or ND (**b**). Ponceau red, Tubulin or Actin were used as loading controls. (**c**–**e**) Breast cancer cells were treated with 10 μM mitoTEMPO 1 h prior to 24 h NR and used for cell death (**c**) and lipid peroxidation (**d**) determination, and Western blot analysis of GPX4 and pH2AX (**e**). Densitometric analysis of GPX4 and pH2Ax immunoreactive bands are reported and normalized to Tubulin loading control. Data are expressed as mean ± S.D. (*n* = 3; * *p* < 0.05, ** *p* < 0.01, *** *p* < 0.001, vs. CTR; ° *p* < 0.05, °°° *p* < 0.0001 vs. NR). Immunoblots reported are representative of three independent experiments for cultured cells and from *n* = 2 out of *n* = 6 mice/group yielding similar results.

Mitochondria play a crucial role in cysteine deprivation-induced ferroptosis and implicate mitochondrial-mediated ferroptosis in tumor suppression [31]. We hence analyzed mitochondrial mass by evaluating the content of some electron transport chain (ETC) subunits. Through Western blot analysis, we disclosed an increase of UQCRC2 (Complex II subunit), MTCO1 (Complex IV subunit) and ATP5A (Complex V subunit) protein content both in MCF7 and MB231 cells treated with NR (Figure 5a). Notably, in tumor explants from the LSAA/HPUFA group, the same ETC subunits were found to be increased along with VDAC1 (Figure 5b), arguing that lipid peroxidation could at least in part be ascribed to increased mitochondrial mass and ETC-derived mitochondrial ROS. To explore whether mitochondrial ROS may play a role in the induction of BC cell death, we treated both MCF7 and MB231 cells with the mitochondrial antioxidant mitoTEMPO prior to NR. This compound significantly mitigated BC cell death (Figure 5c) and prevented the rise in lipid peroxides (Figure 5d). Treatment with mitoTEMPO also mitigated the NR-mediated decrease of GPX4 and the accumulation of pH2AX protein (Figure 5e), finally demonstrating the implication of mitochondria in promoting DNA damage and ferroptosis in BC cells. 

## 3. Discussion

There is strong evidence that many BC cell lines can be induced to ferroptosis by means of genetic or pharmacological approaches [32,33]. Herein, to the best of our knowledge, we demonstrated for the first time that BC cell lines, such as the triple-negative MB231 and the estrogen-receptor-positive MCF7 cells, can be committed to ferroptotic cell death by changing nutrient availability. As matter of fact, using the anti-ferroptosis lipophilic radical-trapping antioxidant FST-1 [34], we were able to significantly mitigate NR-mediated cell death. As a typical marker of ferroptosis, we found a decrease of intracellular cysteine, caused by the lowering of cystine/glutamate antiporter SLC7A11 and likely caused by the decreased cystine amount in the NR culture medium. Being that cysteine is a GSH precursors, the GSH redox state was also successfully affected, with a shift towards pro-oxidant conditions. In parallel, we found a decrease of GPX4, which is an endogenous scavenger of lipid peroxides and hence a key regulator of ferroptosis [2]. The rise in lipid peroxides and accumulation of 4-HNE-protein adducts found in both BC cell lines nicely reflects the significant impairment of the GSH/GPX4 system. Interestingly, we found that BC cells treated with NR are specifically susceptible to SAA lowering, as the only depletion of SAAs nicely recapitulated the effects of NR. Furthermore, we were able to further enhance cell death by the treatment with an exogenous high oxidizable PUFA (i.e., n-6 linoleic acid) and to counteract this event by pre-treating cells with FST-1.

MCF7 and MB231 cells rely on two different metabolic routes, with MB231 being more glycolytic than MCF7 cells, which prefer oxidative phosphorylation for ATP production [35,36]. Moreover, MCF7 cells are autophagy incompetent because of a haploinsufficiency in the gene coding for the autophagy protein Beclin 1 [37]. MB231 cells have the peculiarity to be multidrug resistant, for instance by modulating P-glycoprotein expression [38]. Remarkably, NR or SAA depletion was equally effective in inducing and FST-1 in inhibiting ferroptosis in these cell lines, indicating that these approaches are highly efficient in killing cancer cells in disregard of their metabolic features and resistance to chemotherapeutic drugs. This assumption is in line with evidence that tumor cells, which are resistant to anti-cancer drugs or evade other forms of cell deaths, maintain or acquire sensitivity to ferroptosis [39]. 

Once induced, ferroptosis ameliorates the efficacy of anti-cancer therapies (e.g., chemotherapy, radiotherapy and immunotherapy); hence, combinations with agents targeting ferroptosis signaling is considered a valuable strategy for improving treatment outcomes [2]. Several attempts have been made and are still ongoing to develop ferroptosis-based-cancer therapies that can be translated to clinical practice. The candidate drugs have to be evaluated for their safety; moreover, special attentions should be paid about the dose and the duration of treatment as ferroptosis-inducing drugs, in addition to killing cancer cells, may have off-target toxicity to normal cells and tissues [40]. Safer and efficient therapeutic strategies are hence urgently needed to promote ferroptosis in cancer cells. Overall, the in vitro results demonstrating the susceptibility to ferroptosis of BC cells prompted us to evaluate whether modulating nutrients through diet could be a valuable strategy to regulate ferroptosis markers in vivo as well. PUFAs are highly peroxidizable lipids, and an upregulation of ACSL4, an important isozyme for PUFA metabolism that incorporates them in cell membranes, is required for ferroptosis in BC cells [41]. Actually, this enzyme is downregulated in ferroptosis-resistant and overexpressed in ferroptosis-sensitive cancer cells [42,43]. In light of the results obtained in vitro and in order to induce ferroptosis in vivo, in BC-bearing mice, we used a combined approach that we previously suggested to be promising for anti-cancer therapy based on the metabolic characteristics of cancer cells [19]: (i) we restricted SAAs in order to reduce the efficiency of GSH/GPX4 system; (ii) we increased the level of dietary PUFAs to raise the amount of peroxidizable lipids. Modulation of some ferroptosis hallmarks was obtained in vivo as well, such as the downregulation of NRF2 and GPX4, and the up-regulation of ACSL4. Importantly, our results suggest that concomitant to ferroptosis, BC cells may undergo p53-associated cell cycle arrest, which generally occurs in DNA-damaging conditions that in turn culminate in apoptosis commitment [44]. Accordingly, we found the increase of the DNA damage marker pH2AX, the mRNA up-regulation of p53 down-stream genes and the formation of the PARP-1 cleaved products in BC mass. Considering that both FST-1 and zVAD were not able to completely abrogate death in BC cells, collectively, these results point to the concomitant induction of apoptosis and ferroptosis. Such characteristics of the LSAA/HPUFA diet, i.e., being both pro-ferroptotic and pro-apoptotic, have been also observed for several anti-cancer drugs including Cisplatin and Sorafenib [7,45,46], thus definitely strengthening the anti-BC action of our LSAA/HPUFA diet.

Remarkably, we observed a significant limitation of BC mass growth upon administering the LSAA/HPUFA diet that was accompanied by a prominent decrease of immune cell infiltrates and inflammatory cytokine production within the tumor mass. Angiogenesis is a critical factor in the development of tumors and metastases in numerous cancers. In support of the anti-tumor potential of our LSAA/HPUFA diet, we also disclosed a decrease of vasculature within the explanted BC mass. We can hence postulate that dietary changes function by limiting SAA and enhancing PUFA availability, in addition to committing cells to both apoptosis and ferroptosis, could create an unfavorable environment that limits BC expansion and metastasis. To discover how this dietary strategy has such widespread anti-tumor effects was outside of the scope of our works but merits deeper investigation in the near future. Moreover, in this work, we cannot know whether the LSAA/HPUFA diet has any more effects on tumor engraftment or growth, as it was administered concomitantly with tumor injection. Therefore, experiments in which this diet is administered in established tumors in mice are needed to clarify this issue. 

Proliferation of several cancer cell types depends on glycolysis to ensure an adequate amount of metabolic flux and bioenergetics for macromolecule synthesis and cell division. For this reason, nutrient modulation to reprogram metabolic pathways in cancer cells is considered a promising anti-cancer strategy [36]. Another event that we found in correlation with the induction of apoptotic and ferroptotic cell death was the increase in ETC proteins in both BC cell lines and BC mass in vivo, consistent with a metabolic switch from glycolysis to OXPHOS. This metabolic reprogramming could be specifically harmful for BC cells, since we did not disclose the toxic effects in bio-clinical parameters of mice. Recently, few studies collaboratively indicated the possibility of an involvement of mitochondrial ROS in directing the ferroptotic program. Specifically, the ferroptosis inducers RSL3 (GPX4 inhibitor) and erastin (xCT inhibitor) efficiently promoted mitochondrial ROS production [47,48], and the mitochondrial antioxidant mitoQ prevented ferroptosis [47]. By using the mitochondrial antioxidant mitoTEMPO, we were able to mitigate death in BC cell lines and to completely prevent lipid peroxidation, suggesting that ferroptosis induction largely depends on mitochondria engagement. The implication of mitochondria in orchestrating cell death was confirmed by the recovery of the GPX4 protein and the prevention of pH2AX accumulation upon mitoTEMPO treatment prior to NR.

Our results exceptionally agree with a recent paper that pointed out dietary PUFAs as a selective adjuvant antitumor modality that may efficiently complement pharmacological approaches. In this work, Dierge et al., showed that in cancer cells, incorporated PUFAs undergo peroxidation, thus promoting ferroptosis [17]. Therefore, expanding the anti-cancer effects of our LSAA/HPUFA diet in combination with chemotherapy is of utmost importance to fully exploit its therapeutic potential in BC management. Collectively, our results could suggest future orientation for ferroptosis induction at least in BC cells through dietary strategies. SAAs are found in low amounts in plant foods [49]; therefore, a SAA-deficient diet can be easily designed by increasing the uptake of plant proteins. On the other hand, PUFAs can be administered by augmenting the ratio of PUFA rich foods (e.g., soy, fish, nuts) and/or using commercially available supplements. However, it is absolutely important to balance the PUFA intake and consider the possible side effects of a diet rich in PUFAs as increasing the ratio of n-6 to n-3 PUFAs could lead to inflammation and other health problems (e.g., fatty liver) [50]. 

## 4. Materials and Methods

### 4.1. Cells and Treatments

Human (MCF-7 and MDA-MB-231) BC cell lines were purchased from ATCC-LGC Standards (London, UK) and were cultured in high-glucose Dulbecco’s Modified Eagle’s Medium (DMEM) supplemented with 10% fetal bovine serum (FBS), 100 U/mL penicillin,100 μg/mL streptomycin and 1 mM glutamine (Lonza Sales, Basel, Switzerland). For NR experiments, cells were incubated with Hank’s Balanced Salt Solution (HBSS) containing 1 g/L glucose, 1% FBS and 1% penicillin/streptomycin (Lonza Sales). For SAA depletion, cells were grown in a customized complete DMEM culture medium without cysteine and methionine (Lonza Sales) for 6 or 24 h. To reproduce high PUFA levels, 400 μM of albumin-conjugated linoleic acid (Sigma-Aldrich, St. Louis, MO, USA) was added concomitant to SAA deprivation and maintained throughout the experiment. Albumin-conjugated oleic acid (Sigma-Aldrich) was used at concentration of 400 μM for 24 h. Five μM Ferrostatin-1 (Merck, Darmstadt, Germany) or 10 μM mitoTEMPO (Merck) or 10 μM z-VAD-fmk (Merck) were added 1 h prior to nutrient changes and maintained throughout the experiment. Treatment with erastin (Sigma-Aldrich) was carried out at a concentration of 10 μM for 24 h. Cell death was quantified by trypan blue exclusion.

BALB-neuT mammary cancer cells (H-2d) (TUBO), a cloned line from BALB-neuT mouse mammary gland carcinoma, were kindly provided by Professor G. Forni and Professor F. Cavallo (University of Turin, Turin, Italy) [51] and cultured in DMEM containing 20% FBS, 100 U/mL penicillin and 100 μg/mL streptomycin and 1 mM glutamine (Lonza Sales). Cells were grown at 37 °C in a humidified incubator with an atmosphere of 5% CO_2_. 

### 4.2. Mice and Treatments

BALB/c female mice (*n* = 18, 6–12 weeks old) were subject to overnight fasting prior to TUBO cell inoculation. TUBO cells (5  ×  105) were injected subcutaneously in 200  µL of PBS on the right flank and mice were successively randomly divided in two groups (*n* = 9 mice/group): (1) mice fed with a normal diet (ND, 20P:65C:15F with 12 kcal from cysteine and protein from casein); (2) mice fed with an isocaloric amount of low protein-sulfur amino acid/high polyunsaturated fatty acid diet (LSAA/HPUFA, 7P:48C:45F w/o cysteine and with proteins and fats from soy). The LSAA/HPUFA diet was administered for 1 week and replaced with ND until sacrifice. Mice were maintained one per cage, with a 12 h light/dark cycle, at 23–25 °C. Body weights and tumor masses were measured weekly by a precision scale and caliper, respectively. Mice were sacrificed after 3 weeks in a randomized order to minimize experimental bias. Tumor mass explants were flash-frozen in liquid nitrogen or fixed for immunohistochemical analyses. Investigation has been conducted in accordance with the ethical standards and according to the Declaration of Helsinki and the ARRIVE guidelines. A veterinary surgeon was present during the experiments. The animal care both before and after the experiments was performed only by trained personnel. Mice were bred under pathogen-free conditions in the animal facilities of the University of Rome “Tor Vergata” and handled in compliance with the European Union (EU Directive 2010/63/EU) and institutional standards for animal research. The work was conducted with the formal approval of the local animal care committees (institutional and national), and the animal experiments were registered as legislation requires (Authorization from Ministry of Health no. 187/2016-PR).

### 4.3. Cysteine and Glutathione Determination

Cells were lysed by repeated cycles of freezing and thawing under liquid nitrogen and utilized for HPLC analysis of cysteine, GSH and GSSG, according to Reed et al. [52]. Data are expressed as nanomoles per milligram of proteins. 

### 4.4. Bioinformatic Analysis

To have a comprehensive overview of the effects of nutrient restriction on MCF7 cells, publicly available microarray dataset on Gene Expression Omnibus (https://www.ncbi.nlm.nih.gov/gds, accession on 14 June 2021) under the accession number GSE121378 was analyzed. Differentially expressed genes were selected with a threshold of Log2FC > 0.58 (*p* < 0.05). Functional enrichment analysis including Wikipathways was performed by using the ClueGo plugin of the Cytoscape v3.8 [53]. Default settings were used, except for statistical analysis, which was set at enrichment only, Benjamini-Hochberg correction (adjusted pV < 0.05).

### 4.5. Western Blot Analysis

Tissues or cells were lysed in RIPA buffer (50 mM Tris–HCl, pH 8.0, 150 mM NaCl, 12 mM deoxycholic acid, 0.5% Nonidet p-40, and protease and phosphatase inhibitor cocktail). Ten micrograms of proteins were loaded on SDS–PAGE and subjected to Western blotting. Nitrocellulose membranes were incubated with anti-NRF2 (sc-722, Santa Cruz Biotechnology, Dallas, TX, USA), anti-VDAC1 (sc-8828, Santa Cruz Biotechnology), anti-β-Tubulin (T9026, Sigma-Aldrich, St. Louis, MO, USA), anti-p(S139)H2AX (20E3, Cell Signaling Technologies, Danvers, MA, USA), anti-H2B (sc-515808, Santa Cruz Biotechnology), anti-H3 (sc-10809, Santa Cruz Biotechnology), anti-p21 (sc-397, Santa Cruz Biotechnology), anti-p53 (2527, Cell Signaling Technologies), anti-PARP1 (ALX-210-302, Enzo Life Sciences Inc., Farmingdale, NY, USA), anti-β-Actin (20536-I-AP, Proteintech, Manchester, UK), anti-GPX4 (14432-I-AP, Proteintech), anti-4HNE-adducted protein (AB5605, Millipore Corp., Temecula, CA, USA) (AB5605, Abcam, Cambridge, UK) and anti-Total OxPHOS (MS-604, Abcam, Cambridge, UK) antibodies at 1:1000 dilution.

Appropriate HRP-conjugated secondary antibodies (Bio-Rad Laboratories, Hercules, CA, USA) at 1:3000 dilution were used and immunoreactive bands were visualized by a FluorChem FC3 System (Protein-Simple, San Jose, CA, USA) after incubation of the membranes with ECL Selected Western Blotting Detection Reagent (GE Healthcare, Pittsburgh, PA, USA).

### 4.6. RT-qPCR

RNA was purified by using TRI Reagent (Sigma-Aldrich) and 3 μg was retro-transcripted using MMLV (Promega, Madison, WI, USA). qPCR was performed in triplicate using validated qPCR primers (BLAST), Applied Biosystems™ Power™ SYBR™ Green Master Mix and the QuantStudio3 Real-Time PCR System (Thermo Fisher Scientific, Whaltam, MA, USA). mRNA levels were normalized to RPL8 mRNA and quantified through the 2^−ΔΔCt^ method. The list of primers is reported in Table 1.

### 4.7. Microscope Analysis

Tumor mass explants were embedded in O.C.T., and quickly frozen in liquid nitrogen-cooled isopentane for sectioning at a thickness of 10 μm on a cryostat. Then, slices were processed for immunofluorescence analyses. As primary antibodies, we used anti-S100A9 (89726, Novus biologicals, Littleton, CO, USA), anti-Von Willebrand factor (A0082, Dako-Agilent, Glostrup, Denmark) and anti-p(S139)H2AX (20E3, Cell Signaling Technologies) at 1:200 dilution. Appropriate AlexaFluor-conjugated secondary antibodies (Thermo Fisher Scientific) were used. Hoechst 33342 (Thermo Fisher Scientific) and AlexaFluor 488-conjugated phalloidin (Thermo Fisher Scientific) were used to stain nuclei and actin, respectively, according to manufacturers’ instructions. 

For lipid peroxidation analysis, cells were incubated with C11-BODIPY 581/591 (Thermo Fisher Scientific) at a final concentration of 10 μM for 30 min at 37 °C and fixed with 4% paraformaldehyde solution. Lipid peroxidation was analyzed through fluorescent microscopy. Images were acquired using a Nikon Eclipse TE-2000 fluorescent microscope equipped with UV source and CoolSNAP Photometrics CCD Camera (Nikon Instruments Inc., Amsterdam, The Netherlands).

### 4.8. Cytofluorimetric Analysis and Malondialdehyde Assay

To monitor lipid peroxides and mitochondrial ROS, C11-BODIDY 581/591 and MitoSOX red probes (Thermo Fisher Scientific) were used according to manufacturer’s protocols, respectively. Flow cytometry analyses were performed by Cytoflex and data were analyzed using Cytexpert 2.2 software (Beckman Coulter, Brea, CA, USA). The MDA amounts were measured on 1 mg of tissue by using the lipid peroxidation (MDA) assay kit (MAK085, Sigma-Aldrich) according to the manufacturer’s instructions.

### 4.9. Statistical Analysis

The results are presented as means ± S.D. Statistical analyses were carried out by using the Student’s *t* test to compare the means of two groups. One-way ANOVA was used for comparing the means of more than two groups. Statistical analyses were performed using GraphPad Prism 9 (GraphPad Software Inc., San Diego, CA, USA). In all cases, *p* < 0.05 was set as the significance threshold.

## Figures and Tables

**Table 1:** Human and mouse primers used for RT-qPCR analysis.

*Acsl4 (Mus musculus)*	FW: 5′-AGCCAGCAATAAAGTACACTTACAG-3′ RV: 5′-CAGGGCTCCAAAAGTGACA-3′
*Bax (Mus musculus)*	FW: 5′-TGGAGCTGCAGAGGATGATTG-3′ RV: 3′-AGCCACCCTGGTCTTGGA-3′
*BAX (Homo sapiens)*	FW: 5′-CAGGGTTTCATCCAGGAT-3′ RV: 3′-AAACATGTCAGCTGCCAC-3′
*CDKN1 (Homo sapiens)*	FW: 5′-GTGGCTATTTTGTCCTTGGGC-3′ RV: 3′- GTTCTGACATGGCGCCTGAA-3′
*Cdkn1 (Mus musculus)*	FW: 5′-GCAGAATAAAAGGTGCCACAGG-3′ RV: 3′-AAAGTTCCACCGTTCTCGGG-3′
*GPX4 (Homo sapiens)*	FW: 5′-GCCATCAAGTGGAACTTCACC-3′ RV: 5′-CTTCTCTACTACCAGGGGCTC-3′
*Gpx4 (Mus musculus)*	FW: 5′-ATTGGTCGGCTGCGTGAG-3′ RV: 5′-ACACGAAACCCCTGTACTTATCC-3′
*Il1b* *(Mus musculus)*	FW: 5′-TGCCACCTTTTGACAGTGATG-3′ RV: 5′-AAGGTCCACGGGAAAGACAC-3′
*Il6 (Mus musculus)*	FW: 5′-GGATACCACTCCCAACAGACC-3′ RV: 5′-GCCATTGCACAACTCTTTTCTCA-3′
*Nos2 (Mus musculus)*	FW: 5′-GCCTTCAACACCAAGGTTGTC-3′ RV: 5′-ACCACCAGCAGTAGTTGCTC-3′
*NRF2 (Homo sapiens)*	FW: 5′-TGCCAACTACTCCCAGGTTG-3′ RV: 5′-AAGTGACTGAAACGTAGCCGA-3′
*Nrf2 (Mus musculus)*	FW: 5′-CCTCTGCTGCAAGTAGCCTC-3′ RV: 5′-AATCCATGTCCTGCTGGGACT-3′
*PUMA (Homo sapiens)*	FW: 5′-GAAATTTGGCATGGGGTCTGC-3′ RV: 3′-TCCCTGGGGCCACAAATCT-3′
*RPL8 (Homo sapiens)*	FW: 5′-CACCATGCCTGAGGGTACAA-3′ RV: 5′-CGGGTCTTCTTGGTCTCAGG-3′
*Rpl8 (Mus musculus)*	FW: 5′-GGAGCGACACGGCTACATTA-3′ RV: 5′-CCGATATTCAGCTGGGCCTT-3′
*SLC7A11 (Homo sapiens)*	FW: 5′-CTTTCAAGGTGCCACTGTTCATC-3′ RV: 5′-AGATTGCCAAGATCTCAAGTCCA-3′
*Tnfa (Mus musculus)*	FW: 5′-ATGGCCTCCCTCTCATCAGT-3′ RV: 5′-CTTGGTGGTTTGCTACGACG-3′

## Data Availability

Not applicable.
